# Subjective cognitive impairment and presenteeism mediate the associations of rumination with subjective well-being and ill-being in Japanese adult workers from the community

**DOI:** 10.1186/s13030-021-00218-x

**Published:** 2021-10-02

**Authors:** Kuniyoshi Toyoshima, Masahiko Ichiki, Takeshi Inoue, Akiyoshi Shimura, Jiro Masuya, Yota Fujimura, Shinji Higashi, Ichiro Kusumi

**Affiliations:** 1 Department of Psychiatry, Hokkaido University Graduate School of Medicine, Kita 15, Nishi 7, Kita-ku, Sapporo, 060-8638 Japan; 2 Department of Psychiatry, Tokyo Medical University, 6-7-1 Nishishinjuku, Shinjuku-ku, Tokyo, 160-0023 Japan; 3 Department of Psychiatry, Tokyo Medical University Hachioji Medical Center, 1163, Tate-machi, Hachioji, Tokyo, 193-0998 Japan; 4 Department of Psychiatry, Tokyo Medical University Ibaraki Medical Center, 3-20-1 Chuo, Ami-machi, Inashiki-gun, Ibaraki, 300-0395 Japan

**Keywords:** Rumination, Subjective cognitive impairment, Presenteeism, Subjective well-being, Subjective ill-being, Mediator

## Abstract

**Background:**

In recent years, the roles of rumination, subjective cognitive impairment (SCI), and presenteeism have been emphasized in occupational mental health. However, associations between rumination, SCI, presenteeism, and psychological well-being are not fully understood. We hypothesized that SCI and presenteeism mediate the associations between rumination and subjective well-being (SWB) and subjective ill-being (SIB). Hence, we investigated the mediating roles of SCI and presenteeism in this study.

**Methods:**

A total of 458 adult workers (mean age, 40.8±11.9 years; 44.1% male), who were recruited in Tokyo using convenience sampling, were analyzed in this study. The Ruminative Responses Scale, Cognitive Complaints in Bipolar Disorder Rating Assessment, Work Limitations Questionnaire 8, and Subjective Well-being Inventory were used to evaluate rumination, SCI, presenteeism, and psychological well-being (SWB and SIB), respectively. Path analyses were performed to evaluate the relations between these parameters.

**Results:**

The path analysis indicated that rumination, SCI, and presenteeism were directly and negatively associated with SWB and SIB. Regarding indirect effects, rumination was negatively associated with SWB and SIB via SCI, presenteeism, and both SCI and presenteeism. Furthermore, SCI was negatively associated with SWB and SIB via presenteeism.

**Conclusions:**

The results suggest that SCI and presenteeism mediate the associations of rumination with SWB and SIB in Japanese adult workers. To address the psychological well-being associated with rumination, evaluating SCI and presenteeism simultaneously may be useful in occupational mental health. This study provides key insights into the development of comprehensive intervention strategy based on the biopsychosocial perspective for worker’s psychological well-being.

**Supplementary Information:**

The online version contains supplementary material available at 10.1186/s13030-021-00218-x.

## Background

“Good mental health” is defined as well-being that enables an individual to cope with life stress and work well [1]. In the present study, the concept of psychological well-being contains two independent dimensions—positive and negative affect, in other words, subjective well-being (SWB) indicates a positive state, while subjective ill-being (SIB) indicates a negative state that consists of negative psychological components, such as burnout and tiredness [2]. Notably, the deterioration of SIB does not necessarily lead to the decline of SWB and vice versa [3]. Thus, achieving good mental health should simultaneously be addressed from the viewpoints of SWB and SIB [4]. Furthermore, a recent study has insisted on the need for mental health screening and prevention by general practitioners because the current screening and prevention methods performed thus far may not be sufficient [5]. Indeed, individuals who later develop severe mental illness are rarely identified by current mental health services before they develop the illness, the rate of which is 5%–12% [6, 7]. In occupational mental health, addressing both the SWB and SIB of workers have been urgent issues in recent years.

Presenteeism indicates the state of being physically present at work, although the work productivity can be decreased by illness or other barriers to performance [8]. Presenteeism is considered an important intervention target in occupational mental health [9] because it is closely associated with a worker’s well-being [8, 10]. Various risk factors of presenteeism have been reported, including both physical and mental conditions [11]. Although a systematic review reported the influence of presenteeism on well-being [12], the relationships between the risk factors of presenteeism, SWB, and SIB are not well known.

Good cognitive function occupies a part of good mental health [1]. Cognitive impairment deteriorates psycho-social functioning not only in individuals with psychiatric illnesses but also in the nonclinical adult population [13, 14]. Cognitive function is divided into two categories: objective and subjective cognitive function. Objective cognitive function is primarily evaluated by neuropsychological tests whereas subjective cognitive function is primarily evaluated by self-administered questionnaires [13]. Notably, better subjective cognitive function correlates well with better mental health and higher social functioning [13]. Hence, in recent years, subjective cognitive function has come to be assessed more often in public and occupational mental health [13–15].

Rumination indicates repetitive and passive self-focused thoughts [16] and is positively correlated with objective cognitive dysfunction and depression [17]. Further, perseverative cognition via rumination affects both mental health and somatic symptoms [18]. Recent meta-analyses have highlighted the positive associations among rumination with autobiographical memory specificity [19] and worse executive function [20]. As a result, rumination affects work productivity and mediates the influence of stressful work interruptions on psychosomatic symptoms [21, 22]. Additionally, a previous meta-analysis suggests that rumination is a mediator of the effect of mindfulness-based interventions and affects clinical outcomes [23]. A recent study has suggested the importance of interventions for work-related ruminative thinking [24]. Hence, evaluating rumination has become increasingly important in public health. Regarding the relation between rumination and psychological well-being, rumination predicted deteriorated SWB [25, 26] and SIB in workers [27]. Thus, rumination is considered a predictor of psychological well-being.

Subjective cognitive impairment (SCI) indicates perceived cognitive dysfunction in daily life [28], which is known to act as a mediator in the relation between some clinical parameters. In the general population, SCI mediates the effects of depressive symptoms on psycho-social function [13]. In Japanese adult workers, SCI mediates the association between depressive symptoms and presenteeism [14]. However, whether SCI and presenteeism mediate the associations of rumination with SWB and SIB in Japanese adult workers is not fully understood.

Rumination impacts internal attention switching and predicts SCI [29, 30]. Meanwhile, SCI negatively affects SWB [31] and is strongly associated with psychological distress and predicted deteriorated SIB [32, 33]. Both SCI and presenteeism are more likely to be detected in the workplace [13, 34]. Previous studies have reported that happier workers are more productive at work [35]. Mental conditions, such as depressive and anxiety symptoms, also affect work productivity [11]. Rumination is known to be a characteristic cognition common to depression and anxiety disorders [36]. Furthermore, rumination deteriorates SCI [29, 30], and SCI deteriorates SWB and SIB [31–33]. Therefore, based on these relationships, targeting cognitive dysfunction contributes to promoting psychological well-being [37]. A recent meta-analysis reported the effectiveness of psychological interventions to improve the SWB of workers [38]. Regarding the role of cognitive dysfunction of workers, SCI plays a mediating role in the relationship between insomnia, anxiety, or depressive symptoms and presenteeism [14, 39]. However, whether SCI mediates the relation between rumination and presenteeism is not well known to date.

From the aspect of path model, previous studies suggested the “rumination → cognitive dysfunction” model [40] as well as the “cognitive dysfunction → presenteeism” [14] and “presenteeism → well-being” [41] models. However, the “rumination → cognitive dysfunction → presenteeism → well-being” model has not been investigated; particularly, whether cognitive dysfunction and presenteeism can simultaneously mediate the associations of rumination with SWB and SIB remains unknown.

The theoretical evidence for the present study can be described based on path models. First, rumination predicts SCI, and SCI predicts presenteeism, and presenteeism predicts SWB and SIB: “rumination→SCI” [29, 30], “SCI→presenteeism” [14], “presenteeism→SWB” [42], and “presenteeism→SIB” [43]. Taken together, we set “rumination→SCI→presenteeism→SWB and SIB” models. Second, rumination predicts both SWB and SIB: “rumination→SWB” [25, 26], “rumination→SIB” [27]. Rumination also predicts presenteeism, and SCI predicts SWB and SIB: “rumination→presenteeism” [44, 45], “SCI→SWB” [31], and “SCI→SIB” [32, 33]. Hence, regarding the“rumination→SCI→presenteeism→SWB and SIB” models, the direct effects on our path models were shown in these previous studies. However, the indirect effects of SCI and presenteeism in the relation between rumination and SWB or SIB have not yet been investigated.

Based on the theoretical evidence described above, we hypothesized that SCI and presenteeism mediate the relations between rumination and SWB and SIB. Hence, we performed the mediation analysis for SCI and presenteeism in the present study.

## Methods

### Participants

This cross-sectional study was conducted between April 2017 and April 2018 in Tokyo, Japan, in accordance with the Declaration of Helsinki. At the beginning of this research, the present study design was approved by the Local Ethics Review Board of Tokyo Medical University (Ethics Approval Number: SH3502).

During this period, a total of 597 subjects were recruited. The inclusion criteria were as follows: 20 years of age and older, no severe physical illnesses or brain damage, and agreed to participate in this research. This study obtained written informed consent at the beginning of the assessments. This research was part of a larger study, where several self-administered questionnaires were used [13]. The exclusion criteria of this study were as follows: being unemployed or not working during the assessment and not completing the questionnaires; hence, a total of 139 subjects were excluded and 458 subjects were analyzed in the present study.

### Ruminative Responses Scale (RRS)

The RRS evaluates the severity of ruminative responses. It is composed of 22 items, each of which can be rated using a 4-point Likert scale from 1 (*Almost never*) to 4 (*Almost always*) [46]. The validity and reliability of the RRS have been previously shown, and the RRS has been confirmed to have adequate psychometric properties [47, 48]. In this study, we assessed rumination using the Japanese version [49]. We calculated the RRS total score by summing each score. The higher the total score, the higher the ruminative response.

### Cognitive complaints in bipolar disorder rating assessment (COBRA)

The COBRA evaluates SCI and is composed of 16 items, each of which can be rated using a 4-point Likert scale from 0 (*never*) to 3 (*always*) [28]. The validity and reliability of the COBRA have been shown, and the COBRA has been confirmed to have adequate psychometric properties [28]. The study participants used the Japanese version for which validity and reliability have been previously reported and which has been used in the general adult population [13, 50]. We calculated the COBRA total score by summing each score, with higher scores indicating more serious SCI. Furthermore, a total score ≥15 reflects moderate to severe SCI [13].

### Work Limitations Questionnaire 8 (WLQ-8)

The WLQ-8 evaluates presenteeism. This questionnaire is composed of eight items, each of which is rated using the following Likert scale: *all of the time*, *a great deal of the time*, *some of the time*, *a slight bit of the time*, *none of the time*, and *does not apply to my job* [51–53]. The work productivity loss score, which reflects the % of presenteeism, was calculated by the weighted sum of the scores [14, 54]. Higher scores indicate more severe presenteeism.

### Subjective Well-being Inventory (SUBI)

The SUBI evaluates SWB and SIB, which include 19 and 21 items, respectively [55]. A 3-point Likert scale from 1 to 3 was applied for each item, and the total SWB and SIB scores were calculated by summing the item scores. Higher scores indicate better statuses for both SWB and SIB. The Japanese version, whose reliability and validity have been previously reported, was used in this study [56, 57].

### Statistical analysis

To evaluate the correlations among ruminative responses, SCI, presenteeism, SWB, and SIB, Pearson’s correlation analysis using the Bonferroni method was performed. We performed multiple regression analyses using forced entry to investigate significant predictors of presenteeism, SWB, and SIB. We did not use the goodness-of-fit index because of the saturated models. To show the strengths of the effects, we calculated the standardized path coefficients in the path models. We performed all statistical analyses using STATA/MP 16 (Stata Corp LLC, College Station, TX, USA), except for the path analyses, which were conducted with Mplus version 8.4 (Muthén & Muthén, Los Angeles, CA, USA). For all analyses, the statistically significant level was set as *p* < 0.05.

## Results

### Basic findings

The participants’ characteristics are displayed in Table 1. Nineteen subjects were receiving current psychiatric treatment, and 47 participants had a psychiatric history. Eighty-four participants were determined to have moderate to severe SCI.

**Table 1:** Demographic and clinical characteristics (*N* = 458)

Demographic characteristics	Mean (SD)	***N*** (%)
Age (years)	40.8 (11.9)	
Sex (Male/Female)		202/256 (44.1/55.9)
Married		293 (64.0)
Number of cohabitants	2.9 (1.6)	
Education (years)	14.8 (1.8)	
Currently employed		458 (100)
Psychiatric history		47 (10.3)
Current psychiatric treatment		19 (4.2)
Drinking habit		301 (65.7)
Smoking habit		91 (19.9)
**Clinical assessments**	**Mean (SD)**	***N*(%)**
RRS total score	35.5 (11.2)	
COBRA total score	8.5 (6.6)	
COBRA total score ≥ 15		84 (18.3)
WLQ work productivity loss score	0.04 (0.04)	
SUBI well-being score	38.4 (7.0)	
SUBI ill-being score	51.2 (6.3)	

***Abbreviation***s**:***RRS* Ruminative Responses Scale, *COBRA* Cognitive Complaints in Bipolar Disorder Rating Assessment, *WLQ* Work Limitations Questionnaire, *SUBI* Subjective Well-being Inventory, *SD* Standard deviation.

The outcomes of Pearson’s correlation analysis are shown in Additional file 1; all correlations were statistically significant. Rumination was positively correlated with SCI and presenteeism and negatively correlated with SWB and SIB. SCI was positively correlated with presenteeism and negatively correlated with SWB and SIB. Presenteeism was negatively correlated with SWB and SIB. SWB was positively correlated with SIB.

### Multiple regression analysis

The outcomes of the multiple regression analysis are presented in Table 2. Rumination and SCI were significant positive predictors of presenteeism. Furthermore, rumination, SCI, and presenteeism were significant negative predictors of SWB and SIB.

**Table 2:** Multiple regression analysis (*N* = 458)

	WLQ F (2, 455) = 70.92, *p* < 0.0001		SUBI well-being F (3, 454) = 26.02, *p* < 0.0001		SUBI ill-being F (3, 454) = 97.40, *p* < 0.0001	
**Independent variables**	***β***	***VIF***	***β***	***VIF***	***β***	***VIF***
RRS	0.22^***^	1.18	−0.20^***^	1.24	−0.41^***^	1.24
COBRA	0.36^***^	1.18	−0.12^*^	1.35	−0.26^***^	1.35
WLQ	-	-	−0.17^**^	1.31	−0.11^**^	1.31
**Adjusted*R***^***2***^	**0.23**		**0.14**		**0.39**	

***Abbreviations*:***β* standardized regression coefficients, *VIF* variance inflation factor, *R*^*2*^ Coefficient of determination. ^*^p < 0.05, ^**^p < 0.01, ^***^p < 0.001.

### Path analyses of SWB

**Figure 1:**
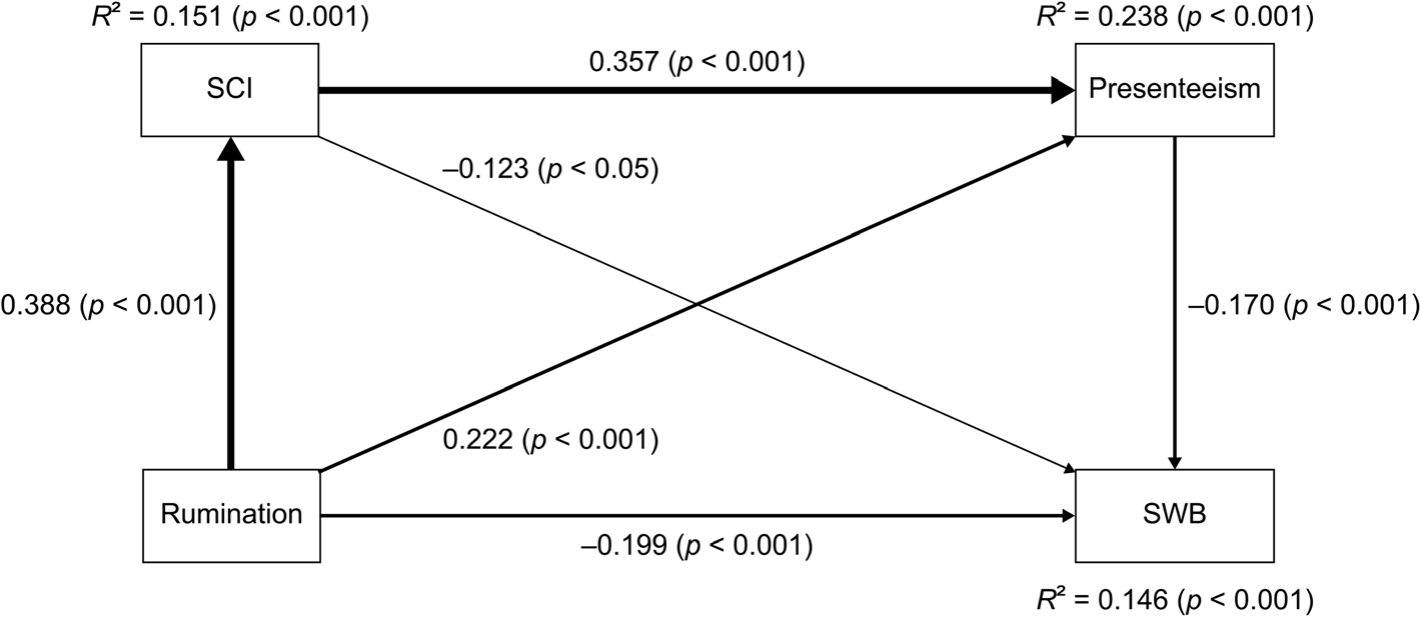
Associations between rumination, SCI, presenteeism, and SWB. The path analysis shows the associations between rumination, SCI, presenteeism, and SWB in 458 adult workers. Direct standardized path coefficients are represented by the values beside the arrows. The strength of the direct effect is demonstrated by the width of the line. Rumination was measured by the RRS score, SCI was measured by the COBRA score, presenteeism was measured by the WLQ work productivity loss score, and SWB was measured by the SUBI well-being score. COBRA, Cognitive Complaints in Bipolar Disorder Rating Assessment; RRS, Ruminative Responses Scale; SCI, Subjective cognitive impairment; SUBI, Subjective Well-Being Inventory; SWB, Subjective well-being; WLQ, Work Limitations Questionnaire; *R*^*2*^, coefficient of determination.

The outcomes of the path analysis of SWB are presented in Table 3 and Figure 1, and all the paths were statistically significant.

**Table 3:** Standardized path coefficients of the path analysis of SWB (*N* = 458)

	Direct effect to		
**From**	COBRA	WLQ	SUBI well-being
RRS	0.388^***^	0.222^***^	−0.199^***^
COBRA		0.357^***^	−0.123^*^
WLQ			−0.170^***^
	**Indirect effect to**		
	COBRA	WLQ	SUBI well-being
RRS		0.139^***^ (via COBRA)	−0.048^*^ (via COBRA)
			−0.038^**^ (via WLQ)
			−0.024^**^ (via COBRA and WLQ)
COBRA			−0.061^**^ (via WLQ)
	**Total indirect effect to**		
	COBRA	WLQ	SUBI well-being
RRS		0.139^***^	−0.109^***^
COBRA			−0.061^**^
	**Total effect to**		
	COBRA	WLQ	SUBI well-being
RRS	0.388^***^	0.361^***^	−0.308^***^
COBRA		0.357^***^	−0.184^***^
WLQ			−0.170^***^

^*^p < 0.05, ^**^p < 0.01, ^***^p < 0.001

There were positive direct effects of rumination on SCI and presenteeism and a negative direct effect on SWB. Secondly, there was a positive direct effect of SCI on presenteeism and a negative direct effect on SWB. Finally, there was a negative direct effect of presenteeism on SWB.

There was a positive indirect effect of rumination on presenteeism via SCI. Second, there were negative indirect effects of rumination on SWB via SCI, via presenteeism, and via SCI and presenteeism. Finally, there was a negative indirect effect of SCI on SWB via presenteeism.

There was a positive total indirect effect of rumination on presenteeism and a negative total indirect effect on SWB. Further, there was a negative total indirect effect of SCI on SWB.

There were positive total effects of rumination on SCI and presenteeism and a negative total effect on SWB. Secondly, there was a positive total effect of SCI on presenteeism and a negative total effect on SWB. Finally, there was a negative total effect of presenteeism on SWB.

The coefficients of determination (*R*^*2*^) of SCI (*R*^*2*^ = 0.151, *p* < 0.001), presenteeism (*R*^*2*^ = 0.238, *p* < 0.001), and SWB (*R*^*2*^ = 0.146, *p* < 0.001) were calculated in the path analysis (Figure 1).

Regarding the mediating effect, SCI mediated the effects of rumination on presenteeism and SWB, and presenteeism mediated the effects of rumination and SCI on SWB. Furthermore, SCI and presenteeism, in this turn, mediated the effect of rumination on SWB.

### Path analyses of SIB

**Figure 2:**
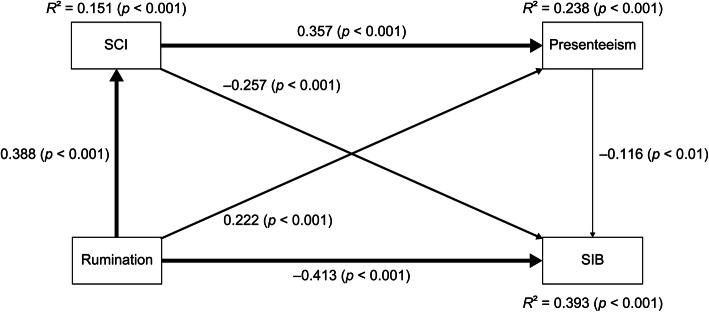
Associations between rumination, SCI, presenteeism, and SIB. The path analysis shows the associations between rumination, SCI, presenteeism, and SIB in 458 adult workers. The direct standardized path coefficients are represented by the values beside the arrows. The strength of the direct effect is demonstrated by the width of the line. Rumination was measured by the RRS score, SCI was measured by the COBRA score, presenteeism was measured by the WLQ work productivity loss score, and SIB was measured by the SUBI ill-being score. COBRA, Cognitive Complaints in Bipolar Disorder Rating Assessment; RRS, Ruminative Responses Scale; SCI, Subjective cognitive impairment; SUBI, Subjective Well-Being Inventory; SIB, Subjective ill-being; WLQ, Work Limitations Questionnaire; *R*^*2*^, coefficient of determination.

The outcomes of the path analysis of SIB are presented in Table 4 and Figure 2, and all paths were statistically significant.

**Table 4:** Standardized path coefficients of the path analysis of SIB (*N* = 458)

	Direct effect to		
**From**	COBRA	WLQ	SUBI ill-being
RRS	0.388^***^	0.222^***^	−0.413^***^
COBRA		0.357^***^	−0.257^***^
WLQ			−0.116^**^
	**Indirect effect to**		
	COBRA	WLQ	SUBI ill-being
RRS		0.139^***^ (via COBRA)	−0.100^***^ (via COBRA)
			−0.026^*^ (via WLQ)
			−0.016^*^ (via COBRA and WLQ)
COBRA			−0.041^**^ (via WLQ)
	**Total indirect effect to**		
	COBRA	WLQ	SUBI ill-being
RRS		0.139^***^	−0.141^***^
COBRA			−0.041^**^
	**Total effect to**		
	COBRA	WLQ	SUBI ill-being
RRS	0.388^***^	0.361^***^	−0.554^***^
COBRA		0.357^***^	−0.298^***^
WLQ			−0.116^**^

^*^p < 0.05, ^**^p < 0.01, ^***^p < 0.001

There were positive direct effects of rumination on SCI and presenteeism and a negative direct effect on SIB. Secondly, there was a positive direct effect of SCI on presenteeism and a negative direct effect on SIB. Finally, there was a negative direct effect of presenteeism on SIB.

There was a positive indirect effect of rumination on presenteeism via SCI. Secondly, there were negative indirect effects of rumination on SIB via SCI, via presenteeism, and via SCI and presenteeism. Finally, there was a negative indirect effect of SCI on SIB via presenteeism.

There was a positive total indirect effect of rumination on presenteeism and a negative total indirect effect on SIB. Further, there was a negative total indirect effect of SCI on SIB. There were positive total effects of rumination on SCI and presenteeism and a negative total effect on SIB. Secondly, there was a positive total effect of SCI on presenteeism and a negative total effect on SIB. Finally, there was a negative total effect of presenteeism on SIB.

The coefficients of determination (*R*^*2*^) of SCI (*R*^*2*^ = 0.151, *p* < 0.001), presenteeism (*R*^*2*^ = 0.238, *p* < 0.001), and SIB (*R*^*2*^ = 0.393, *p* < 0.001) were calculated in the path analysis (Figure 2).

Regarding the mediating effect, SCI mediated the effects of rumination on presenteeism and SIB, and presenteeism mediated the effects of rumination and SCI on SIB. Furthermore, SCI and presenteeism, in this turn, mediated the effect of rumination on SIB.

## Discussion

This study suggests that SCI and presenteeism mediate the associations of rumination with psychological well-being in adult workers. Our findings, which simultaneously report the mediating role of SCI and presenteeism on the relation of rumination with SWB and SIB, are novel.

First, the present study showed that SCI mediated the association between rumination and presenteeism. A previous study reported that SCI mediated the influence of depressive symptoms on presenteeism [14]. Although rumination is positively correlated with objective cognitive impairment and depression [17], the mediator role of SCI on the associations between rumination, depressive symptoms, and presenteeism has not yet been investigated simultaneously. To investigate these relations in greater detail, path analyses using the parameters of rumination, depressive symptoms, SCI, and presenteeism should be performed in the future.

Second, our findings suggest that SCI mediates the association between rumination and SWB. Previous research suggested that SCI mediated the relation between depressive symptoms and quality of life [13]. Furthermore, affective temperaments and personality traits affect SWB [4], and affective temperaments influence SCI [58, 59]. However, our path models did not include affective temperaments or personality traits, which could be a limitation. In the future, the genetic factors should be included in the path models.

Third, our results showed that SCI also mediated the correlation between rumination and SIB. A previous study has suggested that SCI mediates the relation between depressive symptoms and functional disability [13]. Depressive symptoms are positively correlated with rumination and SCI [13, 17], and depressive symptoms mediate the association between affective temperaments and SCI [58]. Furthermore, affective temperaments influence SIB, and SCI mediates the association between affective temperaments and functional disability [4, 59]. Hence, affective temperaments and depressive symptoms could play important roles in the mediating effect of SCI. However, affective factors were not evaluated in this study, which could be a limitation. In the future study, the biological and psychological affective parameters should be included in the path models.

Fourth, in our path models, presenteeism also mediated the associations between rumination, SCI, and SWB and SIB. This is a new finding that suggests the mediating effects of presenteeism on the relations between rumination, SCI, and SWB and SIB. A recent study reported that presenteeism mediated the influence of workaholism on the conflict between work and family [60], and it also mediated the influence of job insecurity on psychological exhaustion [61]. Hence, the mediating role of presenteeism has attracted attention in occupational mental health. However, whether presenteeism mediates the effects of rumination and SCI on work-family conflict or emotional exhaustion is not well known. Furthermore, whether presenteeism mediates the effects of workaholism and job insecurity on SWB and SIB is also not fully understood. In the future, those mediating effects should be investigated in larger longitudinal studies.

Fifth, our findings highlighted the mediating roles of SCI and presenteeism, in this order, on SWB and SIB in adult workers from the community. Hence, to deal with SWB and SIB associated with rumination, evaluating both SCI and presenteeism simultaneously may be useful in occupational mental health. Previous studies reported a “depressive symptoms→ SCI→ presenteeism” model of adult workers [14], and a “depressive symptoms→ SCI→ quality of life” model of the general adult population [13]. Recent study showed a “trait anxiety→ depressive symptoms→ SCI→ SIB” model of the general adult population [62]. The present study suggests a “rumination→ SCI→ presenteeism→ psychological well-being” model of adult workers. Hence, to deal with deteriorated psychological well-being due to rumination, evaluating not only SCI and presenteeism but also depressive symptoms could be useful in occupational mental health.

Sixth, to address the psychological well-being of workers, cognitive behavioral therapy and mindfulness may be effective [38]. Rumination is one of the important factors that can aggravate treatment response for both cognitive behavioral therapy and mindfulness [23, 63]. To address this issue, rumination-focused interventions have recently been developed [64, 65]. Based on our findings and biopsychosocial perspective, the rumination-focused interventions could improve the psychological well-being of workers directly and indirectly via SCI and presenteeism. In the future, we would like to conduct a longitudinal randomized controlled study to test this hypothesis.

Seventh, both SCI and presenteeism correlate well with mental health conditions, including insomnia, depressive symptoms, and state anxiety [14, 39]. According to our findings, both SCI and presenteeism are well associated with rumination and psychological well-being. Furthermore, the mediating roles of SCI and presenteeism in the associations of rumination and psychological well-being are indicated. Therefore, developing early interventions that target both SCI and presenteeism may be needed to deal with the declined psychological well-being due to rumination for adult workers.

Finally, regarding social factors, SCI correlates well with daily living [13] and presenteeism associates well with the working environment [34]. Hence, adjusting both living and working environments could be crucial to improve SCI and presenteeism simultaneously. We suggest that the comprehensive intervention strategy based on the biopsychosocial perspective needs to be developed to deal with psychological well-being associated with rumination in occupational mental health.

### Limitations

This cross-sectional study could not evaluate the cause-and-effect relationships among the variables. We conducted convenience sampling for this exploratory study, which prevents the generalization of our findings. The present study included only workers, which prevents us from generalizing our findings to non-workers. Additionally, all participants in the present study were adults, which precludes the generalization of our findings to adolescents or children. Specifically, this study included only adult workers, which precludes our findings from applying to underage workers. All the participants were Japanese, which prevents generalization of our results to individuals with different cultural backgrounds. The generalization of this study was also limited because study participants were recruited in Tokyo, where many office workers are employed. Hence, the difference between urban and rural areas could not be investigated. The influences of work specialty and workplace support on presenteeism were not assessed [34]. Both the individuals undergoing psychiatric treatment and healthy individuals were investigated together; hence, this heterogeneity could be a limitation. Further, the influence of current mediation was not assessed or controlled for. Finally, memory bias could be a limitation because self-administered questionnaires were used to evaluate clinical characteristics in this study.

## Conclusions

This study suggests that SCI and presenteeism mediate the relation of rumination with SWB and SIB in Japanese adult workers. Hence, evaluating the SCI and presenteeism simultaneously may be useful in occupational mental health. Furthermore, the comprehensive intervention strategy for the deteriorated psychological well-being may need to be developed from the biopsychosocial perspective. Our findings provide key insights into the worker’s psychological well-being.

## Supplementary Information

**Additional file 1: Supplementary Table 1.** Pearson’s correlation analysis using the Bonferroni correction (*N =* 458). Description of data: Associations between rumination, subjective cognitive impairment, presenteeism, and subjective well-being and ill-being. The outcomes of Pearson’s correlation analysis are shown in Additional file 1; all correlations were statistically significant. Rumination was positively correlated with SCI and presenteeism and negatively correlated with SWB and SIB. SCI was positively correlated with presenteeism and negatively correlated with SWB and SIB. Presenteeism was negatively correlated with SWB and SIB. SWB was positively correlated with SIB.

## Data Availability

The datasets used and/or analyzed during the current research are available from the corresponding author upon reasonable request.

## Abbreviations

COBRA: Cognitive Complaints in Bipolar Disorder Rating Assessment
RRS: Ruminative Responses Scale
SCI: Subjective cognitive impairment
SUBI: Subjective Well-Being Inventory
SWB: Subjective well-being
SIB: Subjective ill-being
WLQ: Work Limitations Questionnaire

## References

[1] Fusar-Poli P, Salazar de Pablo G, De Micheli A, Nieman DH, Correll CU, Kessing LV (2020). What is good mental health? A scoping review. Eur Neuropsychopharmacol.

[2] Howell RT, Kern ML, Lyubomirsky S (2007). Health benefits: meta-analytically determining the impact of well-being on objective health outcomes. Health Psychol Rev.

[3] Keyes CLM (2002). The mental health continuum: From languishing to flourishing in life. J Health Soc Behav.

[4] Kanai Y, Takaesu Y, Nakai Y, Ichiki M, Sato M, Matsumoto Y (2016). The influence of childhood abuse, adult life events, and affective temperaments on the well-being of the general, nonclinical adult population. Neuropsychiatr Dis Treat.

[5] Solmi M, Durbaba S, Ashworth M, Fusar-Poli P (2020). Proportion of young people in the general population consulting general practitioners: potential for mental health screening and prevention. Early Interv Psychiatry.

[6] Fusar-Poli P, Rutigliano G, Stahl D, Davies C, Bonoldi I, Reilly T, McGuire P (2017). Development and validation of a clinically based risk calculator for the transdiagnostic prediction of psychosis. JAMA Psychiatry.

[7] McGorry PD, Hartmann JA, Spooner R, Nelson B (2018). Beyond the “at risk mental state” concept: transitioning to transdiagnostic psychiatry. World Psychiatry.

[8] Prochaska JO, Evers KE, Johnson JL, Castle PH, Prochaska JM, Sears LE (2011). The well-being assessment for productivity: a well-being approach to presenteeism. J Occup Environ Med.

[9] Brunner B, Igic I, Keller AC, Wieser S (2019). Who gains the most from improving working conditions? Health-related absenteeism and presenteeism due to stress at work. Eur J Health Econ.

[10] Schmidt B, Schneider M, Seeger P, van Vianen A, Loerbroks A, Herr RM (2019). A comparison of job stress models: associations with employee well-being, absenteeism, presenteeism, and resulting costs. J Occup Environ Med.

[11] Cancelliere C, Donovan J, Stochkendahl MJ, Biscardi M, Ammendolia C, Myburgh C (2016). Factors affecting return to work after injury or illness: best evidence synthesis of systematic reviews. Chiropr Man Therap.

[12] Skagen K, Collins AM (2016). The consequences of sickness presenteeism on health and wellbeing over time: A systematic review. Soc Sci Med.

[13] Toyoshima K, Inoue T, Masuya J, Ichiki M, Fujimura Y, Kusumi I (2019). Evaluation of subjective cognitive function using the Cognitive Complaints in Bipolar Disorder Rating Assessment (COBRA) in Japanese adults. Neuropsychiatr Dis Treat.

[14] Toyoshima K, Inoue T, Shimura A, Masuya J, Ichiki M, Fujimura Y (2020). Associations between the depressive symptoms, subjective cognitive function, and presenteeism of Japanese adult workers: a cross-sectional survey study. Bio Psycho Social Med.

[15] Bhushan A, Fondell E, Ascherio A, Yuan C, Grodstein F, Willett W (2018). Adherence to Mediterranean diet and subjective cognitive function in men. Eur J Epidemiol.

[16] Nolen-Hoeksema S, Wisco BE, Lyubomirsky S (2008). Rethinking rumination. Perspect Psychol Sci.

[17] Kühn S, Vanderhasselt MA, De Raedt R, Gallinat J (2012). Why ruminators won't stop: the structural and resting state correlates of rumination and its relation to depression. J Affect Disord.

[18] Ottaviani C, Thayer JF, Verkuil B, Lonigro A, Medea B, Couyoumdjian A (2016). Physiological concomitants of perseverative cognition: A systematic review and meta-analysis. Psychol Bull.

[19] Chiu CPY, Griffith JW, Lenaert B, Raes F, Hermans D, Barry TJ (2018). Meta-analysis of the association between rumination and reduced autobiographical memory specificity. Memory..

[20] Yang Y, Cao S, Shields GS, Teng Z, Liu Y (2017). The relationships between rumination and core executive functions: A meta-analysis. Depress Anxiety.

[21] Cropley M, Zijlstra FR, Querstret D, Beck S (2016). Is work-related rumination associated with deficits in executive functioning?. Front Psychol.

[22] Zoupanou Z, Rydstedt LW (2019). The mediating and moderating role of affective rumination between work interruptions and well-being. Work..

[23] Gu J, Strauss C, Bond R, Cavanagh K (2015). How do mindfulness-based cognitive therapy and mindfulness-based stress reduction improve mental health and wellbeing? A systematic review and meta-analysis of mediation studies. Clin Psychol Rev.

[24] Cropley M, Collis H (2020). The association between work-related rumination and executive function using the Behavior Rating Inventory of Executive Function. Front Psychol.

[25] Harrington R, Loffredo DA. Insight, rumination, and self-reflection as predictors of well-being. Aust J Psychol. 2011;145(1):39–57. 10.1080/00223980.2010.528072. 10.1080/00223980.2010.528072 21290929

[26] Karabati S, Ensari N, Fiorentino D. Job satisfaction, rumination, and subjective well-being: A moderated mediational model. J Happiness Stud. 2019;20(1):251–68. 10.1007/s10902-017-9947-x.

[27] Liu M, Wang N, Wang P, Wu H, Ding X, Zhao F. Negative emotions and job burnout in news media workers: A moderated mediation model of rumination and empathy. J Affect Disord. 2021;279:75–82. 10.1016/j.jad.2020.09.123. 10.1016/j.jad.2020.09.123 33039777

[28] Rosa AR, Mercadé C, Sánchez-Moreno J, Solé B, Del Mar Bonnin C, Torrentet C (2013). Validity and reliability of a rating scale on subjective cognitive deficits in bipolar disorder (COBRA). J Affect Disord.

[29] Lo BCY, Lau S, Cheung SH, Allen NB. The impact of rumination on internal attention switching. Cognit Emot. 2012;26(2):209–23. 10.1080/02699931.2011.574997. 10.1080/02699931.2011.574997 21614702

[30] van Vugt MK, van der Velde M. ESM-MERGE Investigators. How does rumination impact cognition? A first mechanistic model. Top Cogn Sci. 2018;10(1):175–91. 10.1111/tops.12318. 10.1111/tops.12318 29383884

[31] Schudy A, Żurek K, Wiśniewska M, Piejka A, Gawȩda L, Okruszek L. Mental well-being during pandemic: The role of cognitive biases and emotion regulation strategies in risk perception and affective response to COVID-19. Front Psychiatr. 2020;11:589973. 10.3389/fpsyt.2020.589973. 10.3389/fpsyt.2020.589973 PMC7678487 33240136

[32] Gavelin HM, Boraxbekk CJ, Stenlund T, Järvholm LS, Neely AS. Effects of a process-based cognitive training intervention for patients with stress-related exhaustion. Stress. 2015;18(5):578–88. 10.3109/10253890.2015.1064892. 10.3109/10253890.2015.1064892 26305186

[33] Nelson A, Gavelin HM, Boraxbekk CJ, Eskilsson T, Josefsson M, Järvholm LS, et al. Subjective cognitive complaints in patients with stress-related exhaustion disorder: a cross sectional study. BMC Psychol. 2021;9(1):84. 10.1186/s40359-021-00576-9. 10.1186/s40359-021-00576-9 PMC8132387 34006315

[34] Gosselin E, Lemyre L, Corneil W (2013). Presenteeism and absenteeism: differentiated understanding of related phenomena. J Occup Health Psychol.

[35] Böckerman P, Ilmakunnas P (2012). The job satisfaction-productivity nexus: A study using matched survey and register data. Ind Labor Relat Rev.

[36] Kim S, Yu BH, Lee DS, Kim JH (2012). Ruminative response in clinical patients with major depressive disorder, bipolar disorder, and anxiety disorders. J Affect Disord.

[37] Smith EM, Reynolds S, Orchard F, Whalley HC, Chan SW (2018). Cognitive biases predict symptoms of depression, anxiety and wellbeing above and beyond neuroticism in adolescence. J Affect Disord.

[38] Sakuraya A, Imamura K, Watanabe K, Asai Y, Ando E, Eguchi H (2020). What kind of intervention is effective for improving subjective well-being among workers? A systematic review and meta-analysis of randomized controlled trials. Front Psychol.

[39] Toyoshima K, Inoue T, Shimura A, Uchida Y, Masuya J, Fujimura Y (2021). Mediating roles of cognitive complaints on relationships between insomnia, state anxiety, and presenteeism in Japanese adult workers. Int J Environ Res Public Health.

[40] Fredman Stein K, Morys-Carter WL, Hinkley L (2018). Rumination and impaired prospective memory. J Gen Psychol.

[41] Taloyan M, Aronsson G, Leineweber C, Magnusson Hanson L, Alexanderson K, Westerlund H (2012). Sickness presenteeism predicts suboptimal self-rated health and sickness absence: a nationally representative study of the Swedish working population. PLoS One.

[42] Jeong W, Kim YK, Oh SS, Yoon JH, Park EC. Association between presenteeism/absenteeism and well-being among Korean workers. J Occup Environ Med. 2020;62(8):574–80. 10.1097/JOM.0000000000001901. 10.1097/JOM.0000000000001901 32404833

[43] Zhang J, Wang S, Wang W, Shan G, Guo S, Li Y. Nurses’ job insecurity and emotional exhaustion: The mediating effect of presenteeism and the moderating effect of supervisor support. Front Psychol. 2020;11:2239. 10.3389/fpsyg.2020.02239. 10.3389/fpsyg.2020.02239 PMC7531262 33071851

[44] Vahle-Hinz T, Mauno S, de Bloom J, Kinnunen U (2017). Rumination for innovation? Analysing the longitudinal effects of work-related rumination on creativity at work and off-job recovery. Work Stress.

[45] Zhang J, Li W, Ma H, Smith AP. Switch off totally or switch off strategically? The consequences of thinking about work on job performance. Psychol Rep. 2020:33294120968080. Advance online publication. 10.1177/0033294120968080. 10.1177/0033294120968080 33092480

[46] Nolen-Hoeksema S, Morrow J (1991). A prospective study of depression and posttraumatic stress symptoms after a natural disaster: the 1989 Loma Prieta earthquake. J Pers Soc Psychol.

[47] Treynor W, Gonzalez R, Nolen-Hoeksema S (2003). Rumination reconsidered: A psychometric analysis. Cognit Ther Res.

[48] Schoofs H, Hermans D, Raes F (2010). Brooding and reflection as subtypes of rumination: evidence from confirmatory factor analysis in nonclinical samples using the Dutch Ruminative Response Scale. J Psychopathol Behav Assess.

[49] Hasegawa A (2013). Translation and initial validation of the Japanese version of the Ruminative Responses Scale. Psychol Rep.

[50] Toyoshima K, Fujii Y, Mitsui N, Kako Y, Asakura S, Martinez-Aran A (2017). Validity and reliability of the Cognitive Complaints in Bipolar Disorder Rating Assessment (COBRA) in Japanese patients with bipolar disorder. Psychiatry Res.

[51] Lerner D, Amick BC, Rogers WH, Malspeis S, Bungay K, Cynn D (2001). The work limitations questionnaire. Med Care.

[52] Burton WN, Pransky G, Conti DJ, Chen CY, Edington DW (2004). The association of medical conditions and presenteeism. J Occup Environ Med.

[53] Takegami M, Yamazaki S, Greenhill A, Chang H, Fukuhara S (2014). Work performance assessed by a newly developed Japanese version of the Work Limitation Questionnaire in a general Japanese adult population. J Occup Health.

[54] Lerner D, Chang H, Rogers WH, Benson C, Schein J, Allaire S (2009). A method for imputing the impact of health problems on at-work performance and productivity from available health data. J Occup Environ Med.

[55] Sell H, Nagpal R. Assessment of subjective well-being. The subjective well-being inventory (SUBI). Regional Health Paper. SEARO, 24. New Delhi: Regional Office for South-East Asia, World Health Organization; 1992. https://apps.who.int/iris/handle/10665/204813.

[56] Tonan K, Sonoda A, Ono Y (1995). Production of the subjective well-being inventory, Japanese Edition: it’s reliability and validity. Jpn J Heal Psychol.

[57] Ono Y, Yoshimura K (2001). The subjective well-being inventory Japanese version.

[58] Toyoshima K, Inoue T, Masuya J, Fujimura Y, Higashi S, Kusumi I (2020). Associations among childhood parenting, affective temperaments, depressive symptoms, and cognitive complaints in adult community volunteers. J Affect Disord.

[59] Toyoshima K, Inoue T, Masuya J, Fujimura Y, Higashi S, Kusumi I (2020). Does subjective cognitive function mediate the effect of affective temperaments on functional disability in Japanese adults?. Neuropsychiatr Dis Treat.

[60] Mazzetti G, Vignoli M, Schaufeli WB, Guglielmi D (2019). Work addiction and presenteeism: the buffering role of managerial support. Int J Psychol.

[61] Zheng Z, Peng F, Xu B, Zhao J, Liu H, Peng J (2020). Risk factors of critical & mortal COVID-19 cases: A systematic literature review and meta-analysis. J Inf Secur.

[62] Toyoshima K, Ichiki M, Inoue T, Masuya J, Fujimura Y, Higashi S (2021). The role of cognitive complaints in the relationship between trait anxiety, depressive symptoms, and subjective well-being and ill-being in adult community volunteers. Neuropsychiatr Dis Treat.

[63] Jones NP, Siegle GJ, Thase ME (2008). Effects of rumination and initial severity on remission to cognitive therapy for depression. Cognit Ther Res.

[64] Spinhoven P, Klein N, Kennis M, Cramer AOJ, Siegle G, Cuijpers P (2018). The effects of cognitive-behavior therapy for depression on repetitive negative thinking: a meta-analysis. Behav Res Ther.

[65] Hvenegaard M, Moeller SB, Poulsen S, Gondan M, Grafton B, Austin SF (2020). Group rumination-focused cognitive-behavioural therapy (CBT) *v.* group CBT for depression: phase II trial. Psychol Med.

