# Supplementary material for: Subjective cognitive impairment and presenteeism mediate the associations of rumination with subjective well-being and ill-being in Japanese adult workers from the community
Source: Biopsychosoc Med. 2021 Oct 2;15:15. doi: 10.1186/s13030-021-00218-x (PMC8487485; doi:10.1186/s13030-021-00218-x)
Supplement: Supplementary file 1 — Additional file 1: Supplementary Table 1. Pearson’s correlation analysis using the Bonferroni correction (N = 458). Description of data: Associations between rumination, subjective cognitive impairment, presenteeism, and subjective well-being and ill-being. The outcomes of Pearson’s correlation analysis are shown in Additional file 1; all correlations were statistically significant. Rumination was positively correlated with SCI and presenteeism and negatively correlated with SWB and SIB. SCI was positively correlated with presenteeism and negatively correlated with SWB and SIB. Presenteeism was negatively correlated with SWB and SIB. SWB was positively correlated with SIB. [file 13030_2021_218_MOESM1_ESM.docx]

**Supporting Information**

**Supplementary Table 1. Pearson’s correlation analysis using the Bonferroni correction (*N* = 458)**

|  | RRS | COBRA | WLQ | SUBI well-being |
| --- | --- | --- | --- | --- |
| RRS | - |  |  |  |
| COBRA | 0.39^***^ | - |  |  |
| WLQ | 0.36^***^ | 0.44^***^ | - |  |
| SUBI well-being | −0.31^***^ | −0.28^***^ | −0.30^***^ | - |
| SUBI ill-being | −0.55^***^ | −0.47^***^ | −0.37^***^ | 0.49^***^ |

^***^*p* < 0.001

***Abbreviation***s**:** RRS, Ruminative Responses Scale; COBRA, Cognitive Complaints in Bipolar Disorder Rating Assessment; WLQ, Work Limitations Questionnaire; SUBI, Subjective Well-being Inventory.
